# P02.89. Low Energy Neurofeedback System (LENS) for stress, anxiety, and cognitive function: an exploratory study

**DOI:** 10.1186/1472-6882-12-S1-P145

**Published:** 2012-06-12

**Authors:** S Gillham, H Wild, Z Bayer, M Mitchell, K Sandberg-Lewis, A Colbert

**Affiliations:** 1 Helfgott Research Institute, Portland, USA

## Purpose

Stress and anxiety are endemic problems in western societies and have negative effects on health, wellbeing and cognitive function. Low Energy Neurofeedback System (LENS) is a form of Neurofeedback (NFB) with promising clinical reports supporting its use in addressing these problems. However, research is needed, and this exploratory study was conducted to test compliance; evaluate an innovative blinding procedure and outcome-measure inventories; provide data to calculate sample size and power; and collect preliminary evidence on efficacy of LENS for addressing stress, anxiety and cognitive function in medical students.

## Methods

We utilized a randomized, double-blind, placebo controlled design, and recruited twenty medical students from the National College of Natural Medicine, aged 25-58. Participants were randomized to a series of six LENS (n=10) or sham treatments (n=10) over a period of seven weeks. Exploratory outcome measures included the Perceived Stress Scale (PSS), State and Trait Anxiety Inventory (STAI), Wechsler Abbreviated Scale of Intelligence (WASI), Trail Making Test A and B test (TMT), and the Brown Peterson Task (BPT).

## Results

Of 20 participants, 17 attended all sessions, one missed a single session, and two were dropped from the study. All participants and blinded researchers reported remaining blinded throughout the study. Power analysis of each outcome measure showed greatest sensitivity using the PSS and STAI State form. No significant between group changes were observed with any outcome measures.

## Conclusion

The study design and novel blinding procedure were successful for maintaining participant compliance and blinding. The tests utilized to assess cognitive function are likely not sensitive enough for our study population, which consisted of highly functional individuals. However, the PSS and STAI questionnaires may be sensitive and useful tools for evaluating efficacy of LENS in a future appropriately powered trial. Further study with a larger sample is necessary for assessing efficacy of LENS treatment.

